# A Bengali-Hindi-Telugu parallel corpus for enhanced literary machine translation

**DOI:** 10.1016/j.dib.2026.112775

**Published:** 2026-04-13

**Authors:** Sudeshna Sani, Suryakanth V Gangashetty, Samudravijaya K, Anik Nandi, Aruna Priya, Vineeth Kumar, Akhilesh Kumar Dubey

**Affiliations:** a Department of Computer Science and Engineering, Koneru Lakshmaiah Education Foundation, Guntur, Andhra Pradesh, India; b School of Business, Woxsen University, Hyderabad, Telangana, India; c School of Technology, Woxsen University, Hyderabad, Telangana, India; d Department of Computer Science and Engineering, Faculty of Science and Technology (ICFAI Tech.), ICFAI Foundation for Higher Education (IFHE), Hyderabad, India

**Keywords:** Low-resource Indic languages, Emotion-rich literary text, Cross-lingual embeddings, Semantic similarity, Multilingual corpus construction

## Abstract

Machine translation is an important application in natural language processing. It relies heavily on bilingual and multilingual corpora. India’s rich linguistic diversity has made it challenging to have large and good-quality parallel text corpus for Indian languages. This data article introduces BHT25, a carefully curated parallel corpus of approximately 27,149 trilingual sentence triplets in Bengali, Hindi, and Telugu. The corpus is compiled from emotion-rich literary works of renowned Indian authors such as Rabindranath Tagore and Sarat Chandra Chattopadhyay. Unlike existing datasets that primarily focus on news or government texts, BHT25 emphasizes literary and archaic language varieties, including traditional forms such as Bengali *Sadhu Bhasha*. The development process of BHT25 followed structured data cleaning, preprocessing, sentence alignment, and human validation procedures. Each sentence triplet is assigned a unique identifier to ensure traceability and a systematic analysis. The dataset is released under the CC-BY-4.0 license on Hugging Face, along with preprocessing scripts and documentation, to support research on Indic machine translation and multilingual literary processing. An extended version, annotated with four emotion labels (joy, sadness, anger, fear) and pairwise semantic similarity scores, is also included to support future research on emotion-aware translation and semantic consistency.

Specifications Table

**Table:** 

Subject	Computer Sciences
Specific subject area	Machine translation datasets for Indian literary texts in Bengali, Hindi, and Telugu languages using emotion-rich parallel sentence alignments.
Type of data	**Two versions:** 1. Trilingual Parallel text triplets (base) 2. CSV file with emotion-annotated triplets with semantic scores.
Data collection	Major portion of the parallel sentences were collected from original Bengali literary texts and their Hindi and Telugu translations. Special emphasis was placed on works written in Bengali *Sadhu Bhasha*. The collected data were either digital text or scanned images. The latter were converted into text using Tesseract OCR. The contents include drama dialogues, romantic novels and detective novels among others. A total of 27,149 parallel sentence triplets were gathered. Of these 12,271 sentences (45.2%) were collected from romantic novels and short stories featuring traditional *Sadhu Bhasha* texts written by Rabindranath Tagore and Sarat Chandra Chattopadhyay; 5077 sentences (18.7%) were sourced from news websites and cultural platforms; 4235 sentences (15.6%) were extracted from folk literature containing tales and oral traditions; 3339 sentences (12.3%) were collected from detective stories; 2227 sentences (8.2%) were extracted from the edited ‘Mann Ki Baat dataset’ [1].
Data source location	Institution: Koneru Lakshmaiah Education Foundation Department: Computer Science and Engineering City: Guntur State: Andhra Pradesh Country: India Postal Code: 522,302 **Primary source repositories:** Bengali literary: Rabindra Rachanabali, Project Gutenberg, Archive.org, Tagoreweb, parabass.com, Bidvakolpo etc. Hindi translations: Digital Library of India, Hindi Sahitya, Archive.org, hindikahani, hindi-kavita, Grehalakshmi etc. Telugu translations: Telugu Wikisource, Andhra Digital Library, Sanchika, TeluguPratilipi, Exotic India books, Ravindra Kadavali etc. Government sources: Mann Ki Baat transcripts (pmindia.gov.in) News sources: Anandabazar Patrika, Dainik Jagran, Eenadu, BBC Hindi, Sakshi, Andhra Jyothy.
Data accessibility	**The BHT25 dataset is publicly available without restrictions under CC-BY-4.0 licensing** in **Hugging Face Datasets repository.** Repository name: sudeshna84/BHT25 Data identification number: https://doi.org/10.57967/hf/7426 Direct URL to data: https://huggingface.co/datasets/sudeshna84/BHT25 The complete usage examples, preprocessing scripts, and metadata are available in the README.md file in the repository [2].
Related research article	Sudeshna Sani, Suryakanth V. Gangashetty, Samudravijaya K., Akhilesh Kumar Dubey, “Emotion-Semantic-Aware Neural Machine Translation between Indo-Aryan and Dravidian Languages via Transfer Learning”, in IEEE Access, vol. 14, pp. 4953–4969, 2026, doi: 10.1109/ACCESS.2026.3651419. [3]

## 1 Value of the Data

• These data provide a curated parallel corpus for three major Indian languages - Bengali, Hindi, and Telugu. The dataset supports research in multilingual NLP, low-resource MT, and cross-lingual representation learning.
• The dataset contains structured literary content, including *Sadhu Bhasha*. Such material is difficult to acquire and process due to archaic vocabulary, complex syntax, and OCR challenges. Hence, this corpus a useful resource for literary and culturally grounded text studies.
• The synthetic augmentation methods such as back-translation, often introduce noise in the corpus. But BHT25 contains sentence triplets from renowned novel writers, which were manually checked by human translators. The human expert evaluation ensured high quality of translation. The quality of emotion and semantics were checked via inter-annotator agreement. These annotated data helps researchers who are interested in studying emotional tones and semantic correspondence across languages.
• The dataset allows models to learn emotional, stylistic and culturally grounded patterns in literary texts. It covers a variety of literary genres, such as romantic fiction, short tales, drama, detective narratives, political speeches and news items. This dataset will help research on multilingual alignment, script variation and large language modelling in Indic languages.
• Our publicly accessible Hugging Face repository contains preprocessing scripts, alignment methods, metadata files, and documentation to support reproducibility and dataset extension. These resources allow researchers to adapt the workflow for other language pairs or integrate additional annotations or sources.

## 2 Background

Development of reliable machine translation (MT) systems for a multilingual country like India is not a mere technical requirement but a cultural priority. Consequently, the construction of bilingual or multilingual parallel corpora is essential for training MT models. Despite adapting numerous methods such as web mining, manual collection, and crowdsourcing, the availability of resources for Indic-Indic language pairs remains largely inadequate. Existing national initiatives such as the National Translation Mission [4] and the BhashaDaan project under the National Language Translation Mission [5] highlight this gap. It shows that MT researches in Indic languages mainly favor Hindi-English or Marathi-English pairs, while Bengali-Hindi, Bengali-Telugu, and Hindi-Telugu resources remain comparatively sparse.

Large-scale multilingual collections like Samanantar [6], CCMatrix [7], and other web-mined corpora offer broad language coverage but they provide limited material in literary domains and often lack sentence-level quality verification. Past efforts in building bilingual corpora such as English-Punjabi [8], Chinese-Vietnamese [9], or Uzbek-Kazakh [10] demonstrate continued work toward domain-specific data and hybrid approaches using both manual and automatic method. An example of such an effort for a low-resource language is a compilation of 27,362 Hindi-Kangri parallel sentences [11]. A recent review of parallel corpora for Indic languages [12] further documents the scarcity of high-quality and domain-specific datasets.

Literary translation presents additional challenges beyond limited availability. Many classical Bengali works are written in *Sadhu Bhasha*, a historical version with archaic vocabulary and complex grammar that is stylistically distinct from the contemporary usage. Differences in script, morphology, and sentence structure across Bengali, Hindi, and Telugu also influence alignment and parallelization. Existing parallel datasets like BUET English-Bangla [13] and the IIT Bombay English-Hindi [14] corpora mostly contain news articles, government reports, or everyday conversations. As a result, literary language is not well covered.

To fill this gap, we created BHT25, a Bengali-Hindi-Telugu dataset focused on literary and culturally rich text. This dataset was used in our recent research on neural machine translation for emotion and semantic preservation in Indian languages [3]. A brief description of the dataset is included in that paper. This data article provides a comprehensive description of construction, preprocessing, validation, and annotation of BHT25. This dataset can be used for benchmarking, linguistic studies, dataset comparison, and reproducible MT experiments.

## 3 Data Description

The BHT25 dataset is a parallel text corpus containing 27,149 aligned sentence triplets in Bengali (bn), Hindi (hi), and Telugu (te) languages. The dataset is stored in four column format, with each triplet assigned a unique identifier. The dataset mainly focuses on literary translation, connecting two languages from the Indo Aryan language family (Bengali and Hindi) with a language from the Dravidian language family (Telugu).

### 3.1 Repository structure

The dataset repository is hosted on Hugging Face Datasets platform at https://huggingface.co/datasets/sudeshna84/BHT25 with the following organization:



The BHT25_full.parquet file forms the primary dataset. An expanded version of the dataset with emotions and semantic similarity scores is available in the annotations/directory. The validation/ folder contains human evaluation files for reliability checks. The metadata/ folder provide more information, source references and links to support dataset analysis and reproducibility. Researchers can replicate preprocessing and annotating methods using the python scripts in scripts/ folder. Visual summaries at the dataset level are present in the figures/ directory. Some additional auxiliary files are also included in the repository to support validation and analysis.

### 3.2 Data format and schema

The dataset is provided in Apache Parquet format for efficient storage and processing. The data schema is described in Table 1.

**Table 1:** BHT25 dataset schema.

**Fig. 1:**
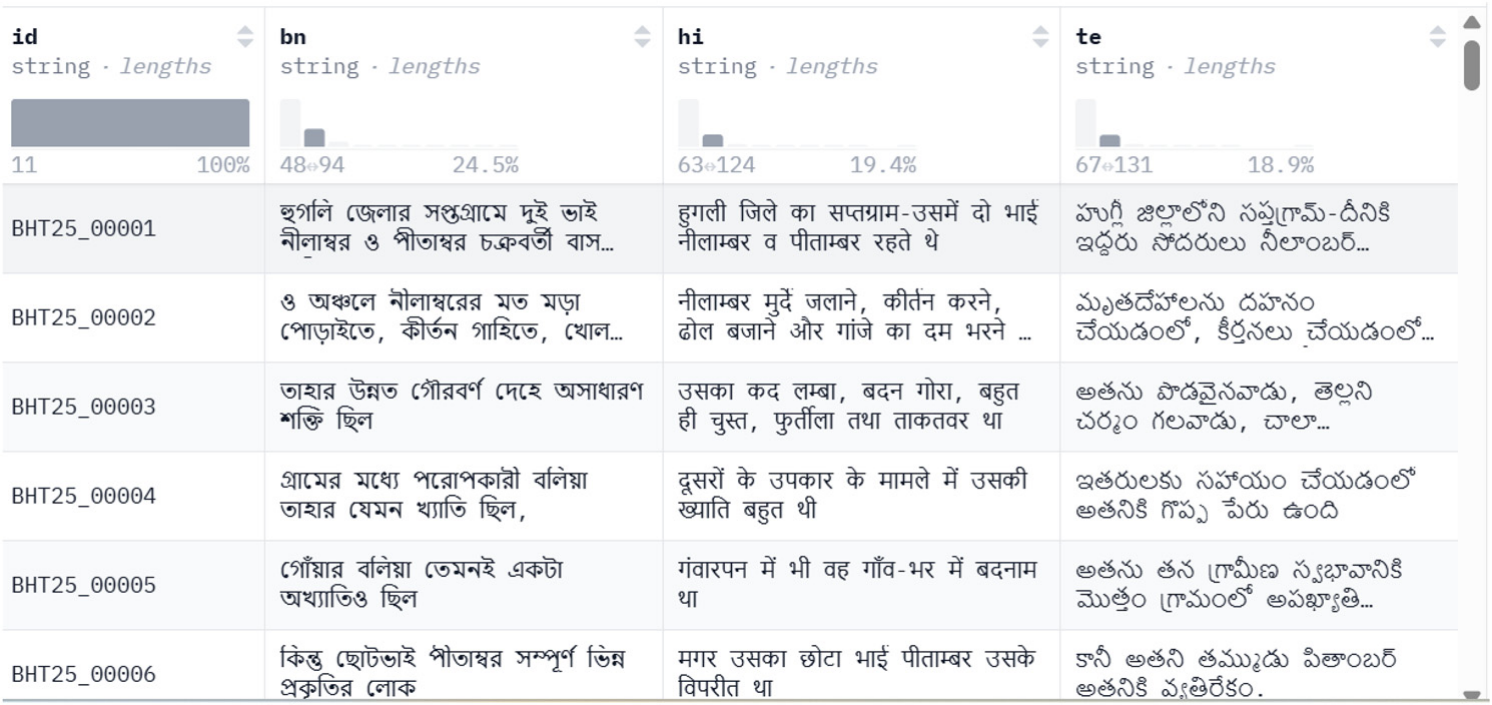
Preview of the BHT25 parquet dataset as displayed on Hugging Face.

The Bengali, Hindi, and Telugu sentence triplets in each row of the dataset constitute a semantically aligned triplet that preserves language-specific stylistic and cultural aspects while conveying the same meaning. The snapshot from the Hugging Face repository in Fig. 1 provides a preview of the dataset's parquet format.

### 3.3 Corpus statistics

The key statistical characteristics of BHT25 corpus are summarized in Table 2.

**Table 2:** Statistical summary of BHT25 Corpus.

Metric	Bengali (bn)	Hindi (hi)	Telugu (te)
Total Sentences	27,149	27,149	27,149
Total Tokens	225,925	299,709	199,462
Vocabulary Size	33,797	23,606	38,944
Average Sentence Length (tokens)	8.3 ± 5.6	11.0 ± 7.7	7.3 ± 5.1
Average Sentence Length (characters)	49.1 ± 35.9	52.5 ± 37.5	54.6 ± 38.9
Minimum Sentence Length (tokens)	1	1	1
Maximum Sentence Length (tokens)	67	122	84
Median Sentence Length (tokens)	7	9	6

**Fig. 2:**
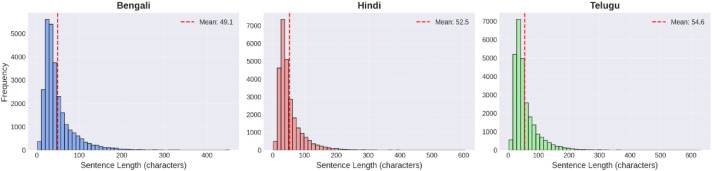
Character level sentence length distribution for three languages.

The distributions of the character level sentence length in the text of the three languages are shown in Fig. 2.

### 3.4 Content characteristics

The corpus encompasses diverse literary genres and writing styles which are listed below.

• Romantic novels and short stories with traditional content (45.2%): 12,271 sentences
• News and cultural content (18.7%): 5077 sentences
• Folk Literature with tales and oral traditions (15.6%): 4235 sentences
• Detective stories (12.3%): 3339 sentences
• Edited ‘Mann ki baat’ dataset (8.2%): 2227 sentences

**Fig. 3:**
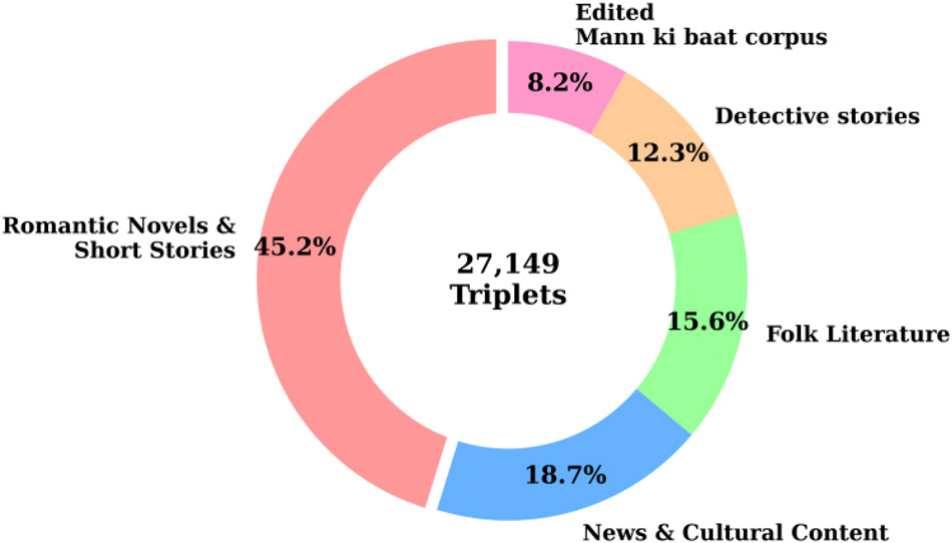
Genre wise distribution of sentences for BHT25 dataset.

Fig. 3 illustrates the diversity of the parallel text corpus.

### 3.5 Language pair characteristics

The dataset supports three primary translation directions as listed in Table 3.

**Table 3:** Language pair properties.

Language Pair	Script System	Word Order	Morphological Type	Primary Challenge
Bengali-Hindi	Devanagari ↔ Bengali	SOV ↔ SOV	Inflectional ↔ Inflectional	Lexical divergence, register variation
Bengali-Telugu	Bengali ↔ Telugu	SOV ↔ SOV	Inflectional ↔ Agglutinative	Morphological complexity, family divergence
Hindi-Telugu	Devanagari ↔ Telugu	SOV ↔ SOV	Inflectional ↔ Agglutinative	Script transfer, compound formation, family divergence

All three language pairs share Subject-Object-Verb (SOV) word order, but differ significantly in morphological strategies and lexical resources. All text data is encoded in UTF-8 to ensure proper representation of Indic scripts.

### 3.6 Data integrity verification

The following checksums assist users in verifying dataset integrity after downloading.

▪ MD5: 1312893c1b4218a046e6bb4fa72eda61
▪ SHA256: a7041d7d03534f1c3afc5d018c4e7745a4a94bdb69b7f2e96b06b952bab3a40a

Users may run checksum verification using the following code

▪ md5sum BHT25_full.parquet
▪ sha256sum BHT25_full.parquet

Successful checksum matching ensures that the files downloaded by users are not corrupted and are suitable for replicating experiments.

### 3.7 Annotated version (Extended version)

The annotations/ folder contain a **second dataset version** with:

▪ Emotion labels for each language
  (0=joy, 1=sadness, 2=anger, 3=fear)
▪ Cross-lingual semantic similarity scores for (bn-hi, bn-te, hi-te) language pairs.

**Fig. 4:**
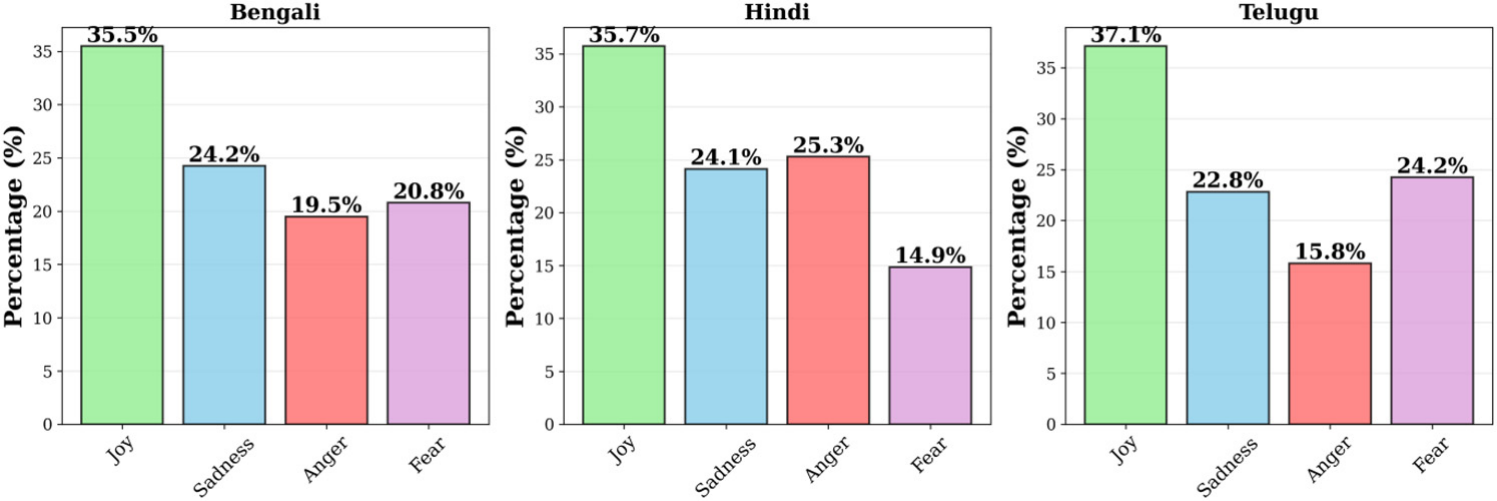
Language wise emotion label distribution across annotated BHT25 sentences.

This extension enables research on emotion-aware translation, style transfer, semantic consistency, and cross-lingual affective analysis. Fig. 4 visualizes relative distribution of emotion labels, assigned automatically by a multilingual emotion classifier (discussed in Section 4.4).

The detailed reports are saved in repository.

## 4 Experimental Design, Materials and Methods

**Fig. 5:**
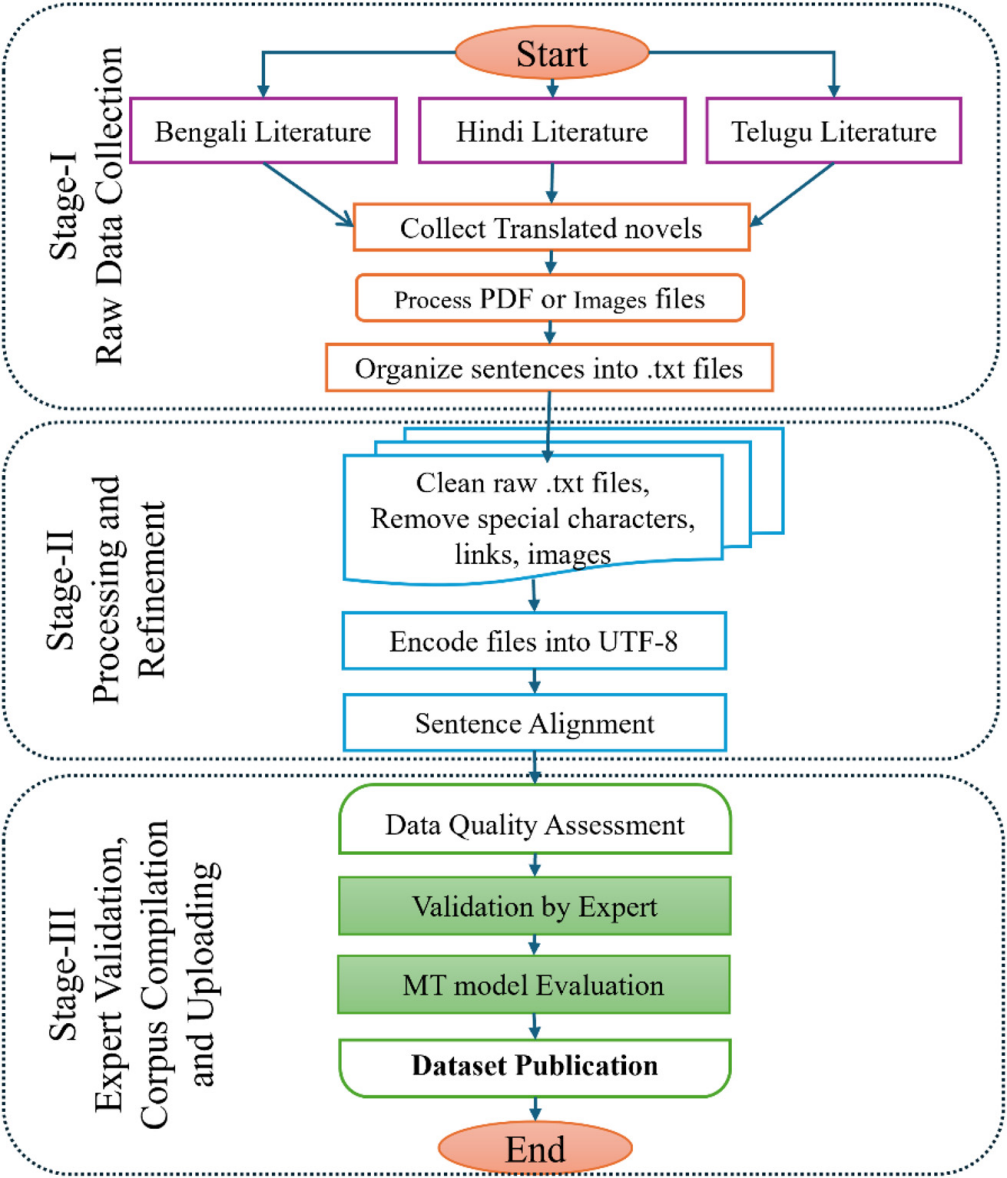
A detailed flow diagram of the process followed in the construction of BHT25 dataset.

We followed a multi-stage pipeline for data collection, preprocessing, alignment, and validation of parallel literary text in Bengali, Hindi, and Telugu languages. The overall workflow consists of three major stages: (i) data collection and organization, (ii) preprocessing and sentence alignment and (iii) dataset validation and publication. A schematic representation of this workflow is provided in Fig. 5.

The 3 major stages of the workflow are described in the following subsections.

### 4.1 Stage I: Data collection and organization

Original Bengali literary texts from novels and stories and their corresponding Hindi and Telugu translated versions were collected from publicly available digital libraries, government archives, and online repositories. The collected material included romantic novels, short stories, drama dialogues, folk literature, and detective fiction. Such old classical Bengali literary works were written in *Sadhu Bhasha*.

Examples of such publicly available works by Rabindranath Tagore and Sarat Chandra Chattopadhyay are *Post Master, Subha, Atithi, IchhaPuran, Kabuliwala, Chhuti, Ginni, Tota Kahini, Udbhar, Sampadak, Swarnamriga, Sasti, Ramkanai-er Nirbuddhita, Musalmanir Golpo, Strir Patra, Pratibeshi, Anadhikar Probesh, Guptadhan, Gora, Biraj Bou, Devdas, Shrikanth, and Detective*.

The collected data consists of 27,149 aligned sentence triplets. The dataset primarily consists of traditional and culturally rooted content. Further, around 2000 sentences (∼8%) were sourced from the publicly available ‘Mann Ki Baat’ dataset [1]. Sentence pairs for Bengali-Hindi and Bengali-Telugu were verified, mapped, and cleaned by linguistic experts using Hindi as a pivot language.

The data sources included both digitally available text and scanned PDF or image documents. Scanned documents were converted into machine-readable text using Tesseract OCR [15]. All collected texts were organized into standardized raw text files prior to preprocessing. A sample of the original source materials, including scanned images and PDF documents are provided in the metadata/Raw_PDF_img_text_files/ folder. The corresponding Python scripts of preprocessing are uploaded in scripts/ folder. These files serve as representative examples of the raw data used during corpus construction.

### 4.2 Stage II: DATA preprocessing and alignment

The raw extracted text contained noise such as headers, footers, page numbers, formatting marks, OCR-mistakes and mixed scripts. We cleaned the text by removing these elements, correcting punctuation and spacing, and splitting the text into one sentence per line using the primary delimiters (|, ?, !,.).

All cleaned files were saved in UTF-8 encoding to preserve script integrity and ensure compatibility across tools. Code-mixed content was handled carefully to maintain language purity. These preprocessing steps resulted in standardized, language-specific sentence files ready for alignment.

After preprocessing, sentence alignment between Bengali-Hindi and Hindi-Telugu was performed using a hybrid method combining sentence length-based traditional statistical method and a semantic similarity cost method. This combination facilitated the resolution of the challenges of aligning sentences across language families with different morphological properties.

#### 4.2.1 Step 1: semantic similarity using cross-lingual word embeddings (CLWE)

We adopted CLWE [16] method to derive semantic relationship between the language pairs. Each sentence in Bengali (BN), Hindi (HI), and Telugu (TE) was mapped into a shared semantic space using the pre-trained *paraphrase-multilingual-MiniLM-L12-v2* [20] model, which produces 384-dimensional embeddings.

For a candidate sentence pair (S_i_,T_j_) semantic similarity is computed using cosine similarity as shown in Eqn. (1).

(1) $$CLWE\_Sim\left(S_{i},T_{j}\right)=\frac{E_{S_{i}}\cdot E_{T_{J}}}{∥E_{S_{I}}∥\cdot ∥E_{T_{J}}∥}\ldots$$

where ${E_{S}}_{i}\;and\;{E_{T}}_{j}$ represent sentence embeddings.

Sentence pairs with similarity scores greater than 0.4 were retained for further processing. This threshold value 0.4 was chosen by testing it on 500 manually aligned sentences.

#### 4.2.2 Step 2: Length-based cost using Gale–Church method

The Gale-Church algorithm [17] exploits the correlation between sentence lengths in source and target languages. The length-based cost is defined as

(2) $$Length\_Cost\left(S_{i},\;T_{j}\right)=\;\frac{{\left(\left|S_{i}\right|-c.\left|T_{j}\right|\right)}^{2}}{\left|S_{i}\right|+\left|\;T_{j}\right|}.\;..\;$$

where $S_{i}\;\text{and}\;T_{j}\;$represent the lengths of the sentences in source and target languages, and ***c*** represents the average length ratio between source and target languages.

#### 4.2.3 Step 3: Enhanced hybrid cost function

To combine semantic and statistical information, an enhanced cost function was defined as:

(3) $$Enhanced\_Cost\left(S_{i},\;T_{j}\right)=\;\alpha \;\cdot \;Length\_Cost\left(S_{i},\;T_{j}\right)+\;\beta \;\cdot \;Semantic\_Cost\left(S_{i},\;T_{j}\right)...$$

where Semantic_Cost is defined as

(4) $$Semantic\_Cost\left(S_{i},\;T_{j}\right)=1-CLWE\_Sim\left(S_{i},T_{j}\right)\ldots$$

In Eq. (3), α=0.6 and β=0.4 represent optimized weighting factors derived through grid search validation on Indic language pairs.

Thereafter, precise threshold values were adopted based on language-specific length ratio across Bengali–Hindi, Bengali–Telugu and Hindi–Telugu pairs. The sentence triplets were retained only if all pairwise alignments satisfied the threshold conditions. This ensured the consistency of meaning across all language pairs.

### 4.3 Stage III: Validation and dataset publication

The aligned sentence triplets were reviewed through a two-tier validation strategy consisting of automated benchmarking and human expert assessment to ensure corpus quality.

#### 4.3.1 Benchmarking with existing models

To validate the utility of BHT25 for neural machine translation research, we report translation performance metrics from our companion publication [3]. In that study, the BHT25 corpus was used to train an Emotion-Semantic-Aware NMT system based on NLLB-200 (No Language Left Behind) [18].

Translation quality was evaluated using standard automatic metrics:

▪ BiLingual Evaluation Understudy (BLEU)
▪ Metric for Evaluation of Translation with Explicit ORdering (METEOR)

Table 4 presents the evaluation scores obtained using the NLLB-200-based model trained exclusively on BHT25. These results demonstrate the dataset's usability for literary machine translation research. Complete experimental methodology, model architecture details, and training procedures are reported in [3].

**Table 4:** Evaluation scores obtained using the NLLB-200 model on BHT25 Dataset (Reproduced from [3]).

Language pair	Translation Quality	
	BLEU	METEOR
Bengali–Hindi	27.6	36.8
Bengali–Telugu	28.1	32.4
Hindi–Telugu	30.41	49.47

#### 4.3.2 Human expert validation

A random sample of 2000 aligned pairs (∼8% of the corpus) was evaluated manually for quality assessment. Selected samples ensured balanced distribution across all five literary genres: narrative (45%), poetry (19%), folk (16%), contemporary (12%), and classical (8%). Alignment quality was also represented, with 35% of pairs having scores above 0.85, 40% between 0.75 and 0.85, 20% between 0.70 and 0.75, and 5% below 0.70. Sentence length diversity was maintained by including short sentences (<10 words, 30%), medium sentences (10–25 words, 50%), and long sentences (>25 words, 20%).

Quality validation was conducted by four expert native speakers (considered as annotators) organized into two language-specific teams.

#### 4.3.3 Annotator profiles

▪ **Bengali–Hindi team (2 annotators with id: BH1, BH2)**: Native Bengali speakers with 12 years of academic and professional experience in literary translation between Bengali and Hindi.
▪ **Hindi–Telugu team (2 annotators with id: HT1, HT2)**: Native Telugu speakers with 10 years of expertise in Telugu–Hindi translation and literary text analysis.

Due to the limited availability of direct Bengali-Telugu bilingual experts, Hindi was used as a pivot language for cross-validation. Bengali-Hindi and Hindi-Telugu alignments were independently verified, and consistency was ensured through the shared Hindi translation.

#### 4.3.4 Evaluation criteria and guidelines

The stratified sample of 2000 sentence triplets was evaluated based on 4 criteria based questions.

▪ **Translation accuracy** (Weight: 30%): *Does the translation convey the same factual content?*
▪ **Semantic equivalence** (Weight: 30%): *Is the intended meaning preserved, including metaphors and cultural context?*
▪ **Grammatical correctnes**s (Weight: 20%): *Is the translated sentence grammatically well-formed and natural?*
▪ **Preservation of literary tone** (Weight: 20%): *Is the original stylistic register and emotional tone maintained?*

Against each sample the annotators assigned ratings on a 5-point Likert scale based on the opinion shown in Table 5.

**Table 5:** Description of the Likert scale score values used for human evaluation.

Score	Description
5	**Excellent**: meaning, grammar, and tone fully preserved
4	**Good**: minor deviations, overall accurate
3	**Acceptable**: understandable with noticeable issues
2	**Poor**: significant errors affecting meaning
1	**Very Poor**: incorrect or unusable translation

#### 4.3.5 Validation procedure

The human annotators were provided with written guidelines describing each evaluation criterion. Initially, all annotators independently evaluated a set of common triplets. Based on differences observed and feedback received, the guidelines were refined to improve clarity and consistency.

After completing the annotation process, the mean rating from all four annotators was calculated for each criterion for every triplet. The composite fluency score was computed as a weighted combination of the four evaluation criteria. Table 6 provides examples of the composite fluency calculation for two sample triplets. For triplet BHT25_00036, the Composite Fluency = (3.50 × 0.30) + (3.63 × 0.30) + (3.50 × 0.20) + (3.50 × 0.20) = 1.05 + 1.09 + 0.70 + 0.70 = 3.54. Within the validation/ folder, the raw evaluation scores assigned by all four human annotators are provided in raw_review_score_data.csv. The aggregated scores, including the computed composite fluency values, are included in expert_quality_scores.csv.

**Table 6:** Review Scores with Descriptive Statistics for Sample Triplets assigned by 4 annotators.

Triplet_id	Annotator id	Translation Accuracy	Semantic Equivalence	Grammatical Correctness	Literary tone Preservation
BHT25_00035	BH1	4	4.5	4	4.5
BHT25_00035	BH2	4	4.5	4	4.5
BHT25_00035	HT1	4	4.5	4	4.5
BHT25_00035	HT2	4	4.5	4	4.5
Mean		4.00	4.5	4.00	4.50
Std. Dev.		0.00	0.00	0.00	0.00
BHT25_00036	BH1	3.5	4	3.5	3.5
BHT25_00036	BH2	3.5	3.5	3.5	3.5
BHT25_00036	HT1	3.5	3.5	3.5	3.5
BHT25_00036	HT2	3.5	3.5	3.5	3.5
Mean		3.50	3.63	3.50	3.50
Std. Dev.		0.00	0.25	0.00	0.00

The overall standard deviation for triplet BHT25_00036 is calculated as:

OverallStdDev=(0.00+0.25+0.00+0.00)/4=0.06

Standard deviation values show the inter-annotator variability across criteria.

#### 4.3.6 Inter annotator agreement

To measure the consistency between reviewers the inter-annotator agreement was computed using Fleiss' Kappa. Kappa was computed separately for each criterion:

○ Translation Accuracy: κ = 0.82
○ Semantic Equivalence: κ = 0.81
○ Grammatical Correctness: κ = 0.84
○ Literary Tone Preservation: κ = 0.83

The overall achieved Kappa score is κ = 0.82. It means that when multiple annotators independently rated the same sentences, they agreed with each other 82% more often than would be expected by random chance. This indicates potential agreement score about evaluation criteria to validate data consistency.

This human evaluation process is used for dataset quality validation and is distinct from the automated annotation process described in Section 4.4.

#### 4.3.7 Publication

Each validated triplet received a unique identifier for traceability. Initially, data is stored in UTF-8 CSV files, then it is serialized into Parquet, and published on Hugging Face under a CC-BY 4.0 license. The data can be accessed by the following link: https://huggingface.co/datasets/sudeshna84/BHT25.

All preprocessing scripts, alignment code, metadata, and documentation are included to support reuse and reproducibility.

### 4.4 Dataset annotation with emotion labels

For extended research purposes, the BHT25.CSV dataset was additionally annotated with emotion labels and pair-wise semantic similarity scores. This annotation process is separate from the human validation described in Section 4.3. Emotion labels were assigned automatically using the MilaNLProc/xlm-emo-t model [19]. It is a multilingual emotion classifier based on the XLM-RoBERTa architecture [19]. This model was used as it is fine-tuned on large-scale multilingual emotion datasets including Bengali, Hindi, and Telugu. The model classifies text into four primary emotion categories: joy (0), sadness (1), anger (2) and fear (3). Pair-wise semantic similarity scores were calculated using the CLWE-based method described in Section 4.1.2. The annotated dataset includes the additional fields such as: emotion_bn, emotion_hi, emotion_te, semantic_bn_hi, semantic_bn_te, semantic_hi_te; these were recorded for all three language pairs. The extended annotated version of this dataset is included in the annotation/ folder of the repository. The corresponding Python script for generating these annotations is included in the scripts/ folder (emotion_semantic_annotation.py).

### 4.5 Software and tools used

The following software, libraries, and tools were used for data processing and validation:

• **OCR:** Tesseract OCR Engine v5.3.0 [15].
• **Programming Language:** Python 3.9.
• **Key Libraries:** sentence-transformers, numpy, pandas, langdetect, scikit-learn.
• **Validation Tools:** Custom scripts for automated checks; spreadsheet software for manual validation tracking.
• **Benchmarking:** Hugging Face Transformers library for NMT model fine-tuning and evaluation.

The complete data processing and validation pipeline was executed on a standard desktop computing environment. All code developed for preprocessing, alignment, and validation will be made available in the associated data repository.

## Limitations

• The BHT25 dataset contains 27,149 sentence triplets, which is useful for literary and academic translation research but smaller than large-scale machine translation corpora containing millions of sentences. This may limit the performance of highly data-intensive neural models.
• The sources are mainly literary works, newspapers, and publicly available archives. This can introduce genre-based patterns and may not fully reflect conversational, technical, or domain-specific language used in everyday communication.
• The dataset currently includes three Indian languages - Bengali, Hindi, and Telugu. While they represent both Indo-Aryan and Dravidian language families, the dataset does not cover other Indian languages, reducing its applicability for broader multilingual or pan-Indian NLP tasks.
• Quality validation involved two native speakers each for Bengali-Hindi and Hindi-Telugu translation pairs. For the Bengali-Telugu pair, validation was indirect and performed through Hindi as a pivot language due to the lack of direct involvement of bilingual experts. This may introduce subjective variability in meaning, fluency, and cultural accuracy.
• The dataset does not include different speaking styles, dialects, or mixed-language usage. So it may not fully match how people actually use multiple languages in everyday life**.**

## Declaration of generative AI and AI-assisted technologies in the writing process

During the preparation of this manuscript, the authors used ChatGPT and Grammarly to improve the language and clarity of the text. The authors carefully reviewed and edited all content generated by these tools and take full responsibility for the final manuscript.

## Ethics Statement

The authors have read and followed the ethical requirements for publication in Data in Brief journal. The current work did not involve human subjects, animal experiments, or any data collected from social media platforms that require informed consent. The dataset was compiled from publicly available and openly accessible sources. No special permissions were required for data collection from these sources. The linguistic experts involved in quality validation are included as contributors via authorship; their contributions represent scholarly collaboration and do not constitute human subject research under the Declaration of Helsinki.

## Credit Author Statement

**Sudeshna Sani**: Conceptualization, Methodology, Data Curation, Software, Writing – Original Draft, Writing - Reviewing and Editing, Visualization, Project Administration; **Suryakanth V Gangashetty**: Supervision, Funding Acquisition, Resources, Writing – Review & Editing; **Samudravijaya K**: Supervision, Methodology, Validation, Writing – Review & Editing; **Anik Nandi**: Data Curation, Validation, Investigation, Resources (Linguistic Expertise); **Aruna Priya**: Data Curation, Validation, Investigation, Resources (Linguistic Expertise); **Vineeth Kumar**: Data Curation, Validation, Investigation, Resources (Linguistic Expertise); **Akhilesh Kumar Dubey**: Software, Visualization, Writing – Review & Editing, Project Administration.

## Data Availability

Hugging FaceBHT25: Bengali-Hindi-Telugu Parallel Corpus for Literary Machine Translation (Original data).
